# Supplementary material for: Identification of Dysregulated microRNAs in Glioblastoma Stem-like Cells
Source: Brain Sci. 2023 Feb 18;13(2):350. doi: 10.3390/brainsci13020350 (PMC9953941; doi:10.3390/brainsci13020350)
Supplement: Supplementary file 1 [file brainsci-13-00350-s001.zip › brainsci-2124871-supplementary.pdf]

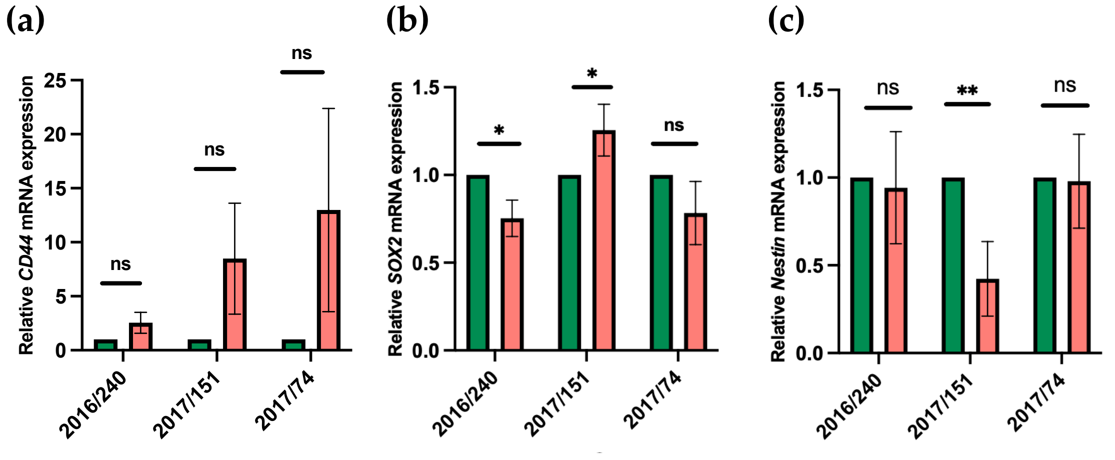

Supplementary Figure S1. Further characterization of GSCs and differentiated astrocytic tumor cells. Relative mRNA expression levels of *CD44* (a), *SOX2* (b), and *Nestin* (c) were calculated via RT-qPCR. Green Bars represent the GSC lines, and differentiated astrocytic tumor cells are shown as red bars. For CD44, two, for SOX2 and Nestin, three independent experiments were performed in triplicates. Mean values +/- SD are shown. Paired students *t-tests* were applied to determine significance with ns $p > 0.05$, * $p < 0.05$, and ** $p < 0.01$.

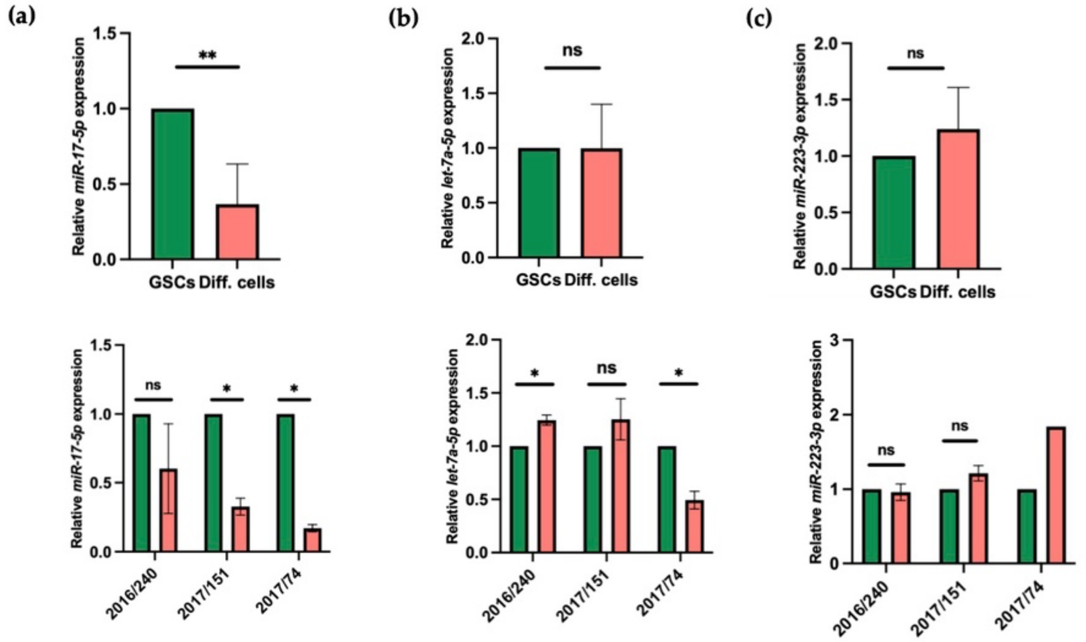

Supplementary Figure S 2. Verification of three dysregulated miRNAs in three GSC-lines. (a) MiR-17-5p is upregulated in all three GSC-lines (indicated in green) in comparison with differentiated cells lines (indicated in red). UniSp6 was used for normalization. Two independent experiments were conducted. **(b)** Let-7a-5p expression in three GSC-lines (green bars) and corresponding differentiated, astrocyte cells (red bars). Two independent experiments were conducted. miR-24-5p was used as a housekeeping miRNA. **(c)** MiR-223-2p expression in GSCs (green) and differentiated tumor cells (red). In case of 2016/240 and 2017/151 two independent experiments were conducted and in case of 2017/74 results of one experiment are demonstrated. miR-24-5p was used as a housekeeping miRNA. Results are given as mean +/- SD. A paired students t-test was applied to determine significance: ns $p > 0.5$, * $p < 0.05$, and ** $p < 0.01$.
